# Rare secondary pituitary abscess arising in a craniopharyngioma: A case report and literature review

**DOI:** 10.3892/etm.2025.12908

**Published:** 2025-06-19

**Authors:** Rui Fan, Runsheng Zhao, Yan Zhong, Weiqing Wan

**Affiliations:** 1 Department of Neurosurgery, Beijing Tiantan Hospital, Capital Medical University, Beijing 100071, P.R. China; 2 China National Clinical Research Center for Neurological Diseases, Beijing 100071, P.R. China

**Keywords:** craniopharyngioma, pituitary abscess, secondary infection, parasellar

## Abstract

A pituitary abscess is an extremely rare condition, classified as either primary or secondary. Secondary pituitary abscesses can arise from pre-existing pituitary lesions such as craniopharyngiomas. The present study describes the case of a 59-year-old man with a secondary pituitary abscess originating from a craniopharyngioma, presenting with a 10-month history of progressive visual decline, dizziness, headaches, nausea and vomiting. Although there were no overt endocrine symptoms, hormonal evaluation revealed hypothyroidism and growth hormone axis suppression, along with elevated prolactin levels due to pituitary stalk effect. MRI showed chronic inflammation in the sellar and suprasellar regions. The patient was initially treated with antibiotics, but symptoms persisted, necessitating craniotomy. Intraoperatively, a cystic structure was incised, releasing white purulent material. Histopathology confirmed a papillary craniopharyngioma with marked inflammatory infiltration. Postoperatively, the patient showed clinical improvement with antibiotic therapy; however, residual pituitary insufficiency and mild diabetes insipidus required long-term management. This case underscores the importance of considering secondary pituitary abscesses in the differential diagnosis of sellar lesions with atypical presentations. It is one of the few reported cases of a pituitary abscess secondary to a papillary craniopharyngioma, highlighting the critical role of timely surgical intervention and the diagnostic value of histopathological confirmation. The publication of this case provides new insights into the diagnostic challenges and management strategies for this rare but life-threatening condition.

## Introduction

Pituitary abscesses are extremely rare in clinical practice, accounting for ~1% of all pituitary lesions (1). However, as a central nervous system infection, they pose a risk of disability and mortality (2). Pituitary abscesses are classified as primary or secondary. Primary pituitary abscesses are more common, accounting for ~67% of cases, and typically result from hematogenous bacterial spread to an otherwise normal pituitary gland. By contrast, secondary pituitary abscesses, which arise from pre-existing sellar lesions such as pituitary adenomas, Rathke's cleft cysts or craniopharyngiomas, represent 33% of cases (3). The pathogenetic mechanisms leading to secondary pituitary abscesses include anatomical distortion, impaired circulation and altered immune status, which predispose patients to infection (4). The clinical presentation of pituitary abscesses is variable, with symptoms including hormonal imbalances, diabetes insipidus and visual disturbances, often due to mass effects. When the pituitary gland is infected, hypopituitarism may occur, resulting in deficiencies in one or more pituitary hormones. This dysfunction can cause severe endocrine disorders, such as diabetes insipidus (due to antidiuretic hormone deficiency), hypothyroidism (due to thyroid-stimulating hormone deficiency) and adrenal insufficiency (due to adrenocorticotropic hormone deficiency) (5). These hormonal imbalances can lead to systemic complications, including electrolyte disturbances, metabolic abnormalities and cardiovascular instability, emphasizing the potentially dangerous nature of pituitary abscesses (6). MRI findings typically reveal a cystic lesion with ring enhancement, which is hyperintense on T2-weighted images and hypointense on T1-weighted images. However, despite advancements in imaging technology, the preoperative diagnosis of pituitary abscesses remains challenging, particularly in cases associated with pre-existing sellar lesions such as craniopharyngiomas. Early surgical drainage combined with antibiotic therapy remains the cornerstone of treatment, but outcomes depend heavily on a timely diagnosis and the extent of the underlying lesion. Delayed treatment may result in irreversible hormonal deficiencies and increased morbidity (7).

The difficulty in diagnosing secondary pituitary abscesses, particularly those associated with craniopharyngiomas, necessitates a broader understanding of their pathophysiology and a more systematic approach to diagnosis and management. Early recognition and intervention are crucial for reducing the high prevalence of pituitary dysfunction in patients treated for these abscesses and for improving overall prognosis. The present case report highlights the clinical features and management of a secondary pituitary abscess arising from a craniopharyngioma and provides a literature review of similar cases.

## Case report

A 59-year-old man presented to Beijing Tiantan Hospital, Capital Medical University (Beijing, China) in August 2023 with complaints of progressive visual deterioration in both eyes, along with visual field constriction, dizziness, headaches, nausea, vomiting and an intermittent fever with a peak temperature of 39.2˚C. The visual impairment had worsened over several months, with difficulty recognizing faces and reading, particularly in dim lighting. The patient reported a gradual narrowing of the peripheral vision, which was later confirmed as bitemporal hemianopsia, a hallmark of sellar region pathology. The patient also described intermittent diplopia and a sensation of 'pressure' behind the eyes. In addition, a single episode of transient loss of consciousness accompanied by convulsions lasting about 1 min was experienced. Neurologically, the patient exhibited mild confusion, generalized fatigue and occasional dizziness upon standing, raising suspicion of an underlying endocrine disturbance. The patient did not have a family history of cancer.

An MRI scan performed at a local hospital revealed a cystic lesion with rim enhancement in the sellar and suprasellar regions, measuring ~18x22x20 mm, with mass effect on the optic chiasm. The lesion exhibited heterogeneous hyperintensity on T2-weighted images and hypointensity on T1-weighted images, suggesting a pituitary abscess (data not shown). The patient was treated with intravenous cephalosporin antibiotics; however, the symptoms persisted, and the patient was subsequently transferred to the Department of Neuroinfections and Immunology at Beijing Tiantan Hospital (Beijing, China).

**Figure 1:**
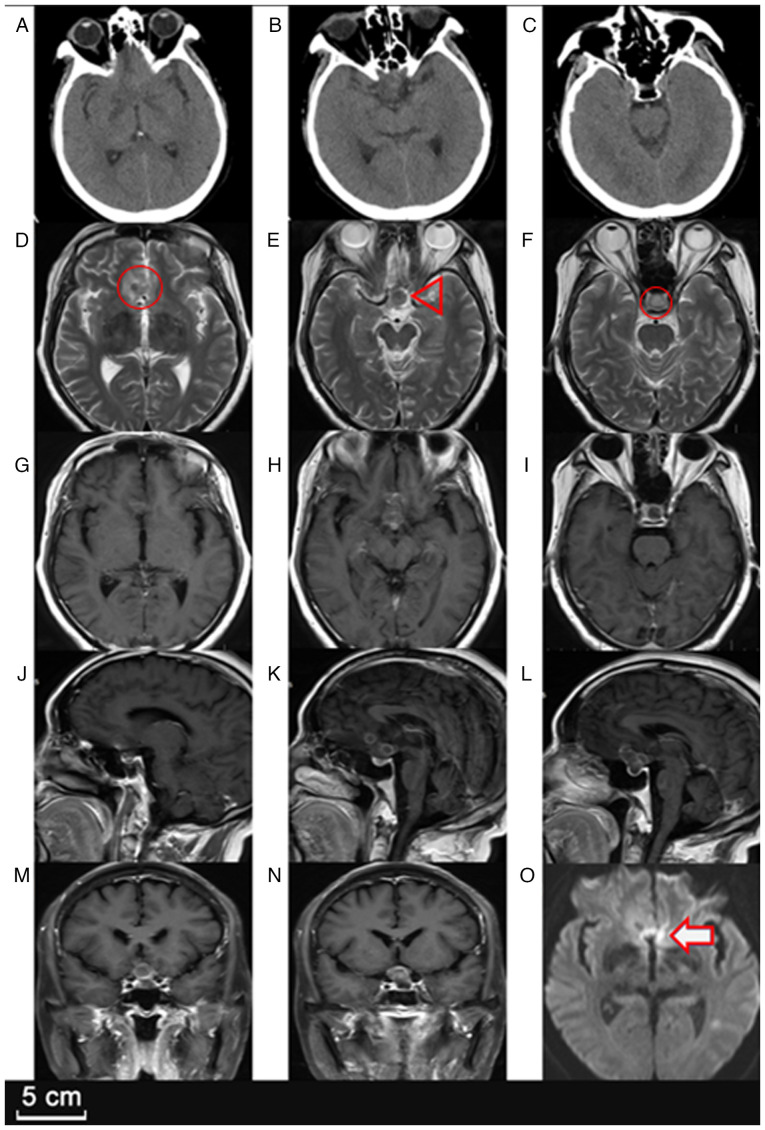
First imaging examination in Beijing Tiantan Hospital. (A-C) CT scans showing uneven sellar density. (D) Axial T2-weighted MRI showing irregular mixed signal intensity in the suprasellar and right frontal lobe areas (lesion indicated by a circle). (E) Axial T2-weighted MRI revealing that the lesion compresses the optic chiasma (indicated by an arrowhead), correlating with visual impairment. (F) Axial T2-weighted MRI showing mixed signals in the sellar region (indicated by a circle). (G-N) Axial, sagittal and coronal T1-weighted MRI images revealing annular enhancement of the saddle region and the right frontal lobe. (O) Axial diffusion-weighted MRI with a high signal intensity in the lesion area (indicated by an arrow), indicating limited diffusion in the area.

Neurological examination upon admission, 5 days after the patient initially presented to the local hospital, revealed bilateral visual decline with confirmed bitemporal hemianopsia. The visual acuity was significantly reduced, measured at 0.2 (right eye) and 0.1 (left eye) on the Snellen chart. Further imaging studies, including a head CT scan and contrast-enhanced MRI (Fig. 1), indicated a well-defined, multilobulated, ring-enhancing lesion in the sellar region, extending into the suprasellar cistern. Additionally, another small ring-enhancing lesion was noted in the right frontal lobe, suggesting the possibility of multifocal abscess formation. The sellar lesion showed mild perifocal edema, with compression of the optic chiasm and partial displacement of the third ventricle, raising concern for obstructive hydrocephalus.

A comprehensive hormonal evaluation of the pituitary gland (Table I) revealed significant suppression of the thyroid and growth hormone axes, with a free thyroxine level of 7 pmol/l (reference range, 7.64-16.03 pmol/l) and an insulin-like growth factor 1 level of 33.7 ng/ml (reference range, 45-210 ng/ml). Additionally, the patient exhibited symptoms of adrenal insufficiency, including postural dizziness and chronic fatigue, with a morning cortisol level of 3.1 µg/dl (reference range, 5-25 µg/dl), necessitating corticosteroid replacement. Oral prednisone acetate tablets were administered at a daily dose of 10 mg from the time of diagnosis of adrenocortical insufficiency until postoperative hormone replacement therapy. The dosage was gradually tapered and eventually discontinued after follow-up revealed progressive recovery of pituitary function. Prolactin was elevated to 54.4 ng/ml (reference range, 2.50-17.00 ng/ml) due to the pituitary stalk effect. Urine tests revealed low urine specific gravity (1.002) and a marked increase in 24-h urinary sodium excretion (609.96 mmol/day), supporting a diagnosis of central diabetes insipidus. The patient underwent a lumbar puncture, and the cerebrospinal fluid (CSF) analysis revealed a markedly elevated white blood cell count of 1,301/µl (reference range, 0-8/µl), with 70.4% polymorphonuclear cells, consistent with a bacterial infection. CSF protein was elevated at 251.25 mg/dl (reference range, 15.00-45.00 mg/dl) and lactate level was increased to 3.8 mmol/l (reference range, 1.1-2.4 mmol/l), suggesting an ongoing central nervous system (CNS) infection.

### Initial treatment and disease progression

**Figure 2:**
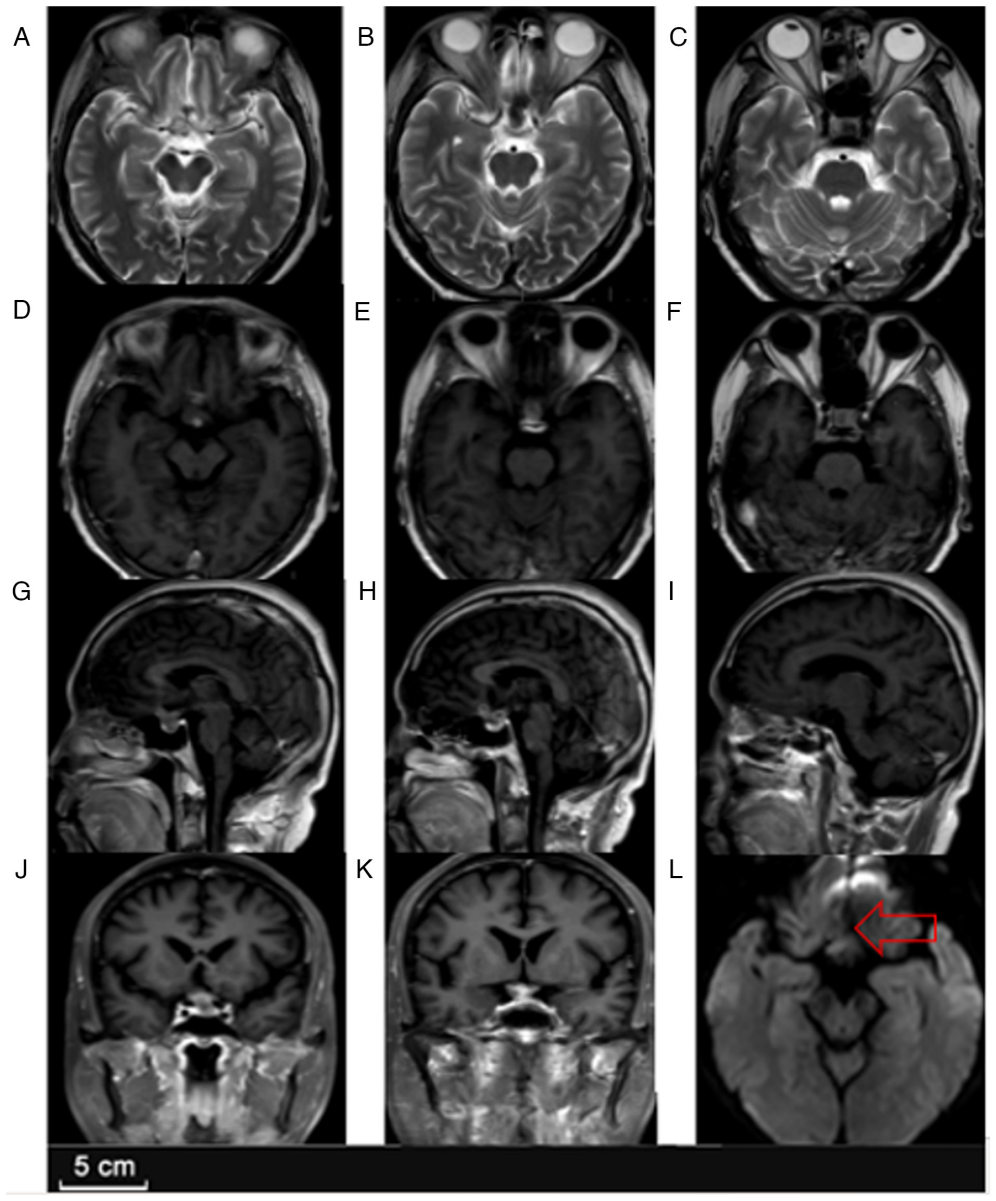
Follow-up MRI at discharge. (A-C) Axial T2-weighted MR images demonstrating reduced lesion size and decreased perilesional edema in both the sellar and suprasellar regions. (D-F) Axial T1-weighted contrast-enhanced MR images revealing a slightly reduced annular enhancement in the sellar and right frontal lobes, indicating a mild regression of the lesion compared to prior imaging. (G-I) Sagittal T1-weighted contrast-enhanced MR images showing reduced enhancement in the sellar and suprasellar regions, with a smaller extent of involvement in the right frontal lobe. (J and K) Coronal T1-weighted contrast-enhanced MR images demonstrating decreased ring-like enhancement and lesion size in both the sellar and right frontal areas. (L) Axial diffusion-weighted MRI showing a mild high signal in the sellar region (indicated by an arrow), suggesting slight diffusion restriction.

Empirical broad-spectrum antibiotic therapy with meropenem (1,000 mg, bid, iv, one month) and vancomycin (500 mg, every day, intravenously for 1 month) was initiated to control the CNS infection, along with oral levetiracetam (500 mg, twice per day, orally; administered long-term and continued postoperatively for prophylactic purposes) for seizure control and desmopressin (0.1 mg, three times a day, orally; administered long-term until the symptoms of diabetes insipidus gradually resolved after surgery) for central diabetes insipidus. The patient's fever and systemic inflammatory response improved, and the patient's body temperature returned to normal. However, despite a prolonged course of intravenous antibiotics, the visual deficits and endocrine dysfunction persisted. Follow-up MRI (Fig. 2) at discharge in January 2024 showed a slight reduction in lesion size but persistent enhancement and perifocal edema, indicating that infection had not been completely eradicated.

After discharge, the patient continued antibiotic treatment at a community hospital. However, in March 2024, the fever, dizziness and lethargy recurred, and the patient again had a seizure episode characterized by jaw clenching, loss of consciousness and generalized tonic-clonic movements lasting ~1 min.

### Surgical intervention

The patient was readmitted in April 2024 to Neurosurgical Oncology Unit 7 at Beijing Tiantan Hospital. A physical examination upon admission revealed that the patient was conscious but lethargic, with further decreased visual acuity (0.1 in both eyes), constricted visual fields and persistent endocrine symptoms. Neck stiffness was present, raising suspicion of worsening intracranial infection.

**Figure 3:**
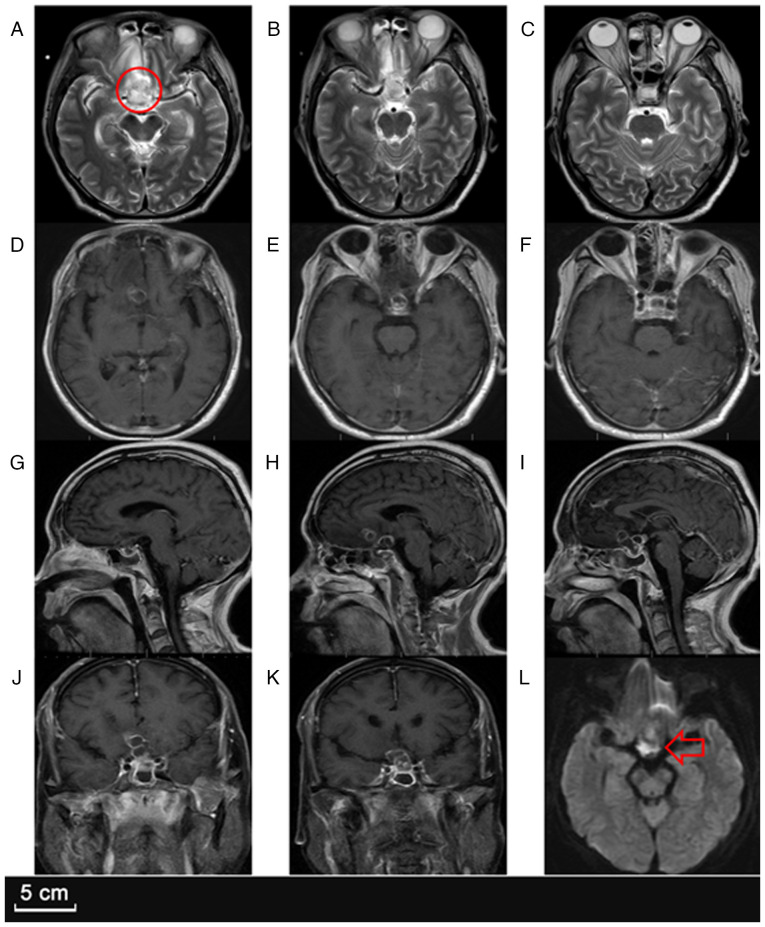
Preoperative MRI. (A) Axial T2-weighted MRI showing a larger area of mixed signal intensity in the sellar region (indicated by a circle) compared with the initial scan. (B and C) Axial T2-weighted MR images demonstrating increased perilesional edema and lesion expansion in both the sellar and suprasellar regions. (D-F) Axial T1-weighted contrast-enhanced MR images revealing annular enhancement in the sellar and frontal lobes, with an enlarged enhancement range compared to previous imaging. (G-I) Sagittal T1-weighted contrast-enhanced MR images showing progressive enhancement in the sellar and suprasellar regions as well as extension into the right frontal lobe. (J and K) Coronal T1-weighted contrast-enhanced MR images displaying more extensive ring-like enhancement in the sellar and right frontal areas. (L) On axial diffusion-weighted MR imaging, the saddle region (indicated by an arrow) exhibits a pronounced signal, suggesting evident limitations in dispersion.

A follow-up MRI (Fig. 3) demonstrated marked enlargement of the sellar lesion, which had increased to 26x32x28 mm, with intensified ring enhancement, worsening perifocal edema and an increased mass effect on the optic chiasm. The right frontal lesion had also slightly enlarged, suggesting ongoing infection despite antibiotic therapy. Given the failure of conservative management, the neuro-oncology team decided to proceed with a craniotomy for lesion resection and thorough abscess drainage.

**Figure 4:**
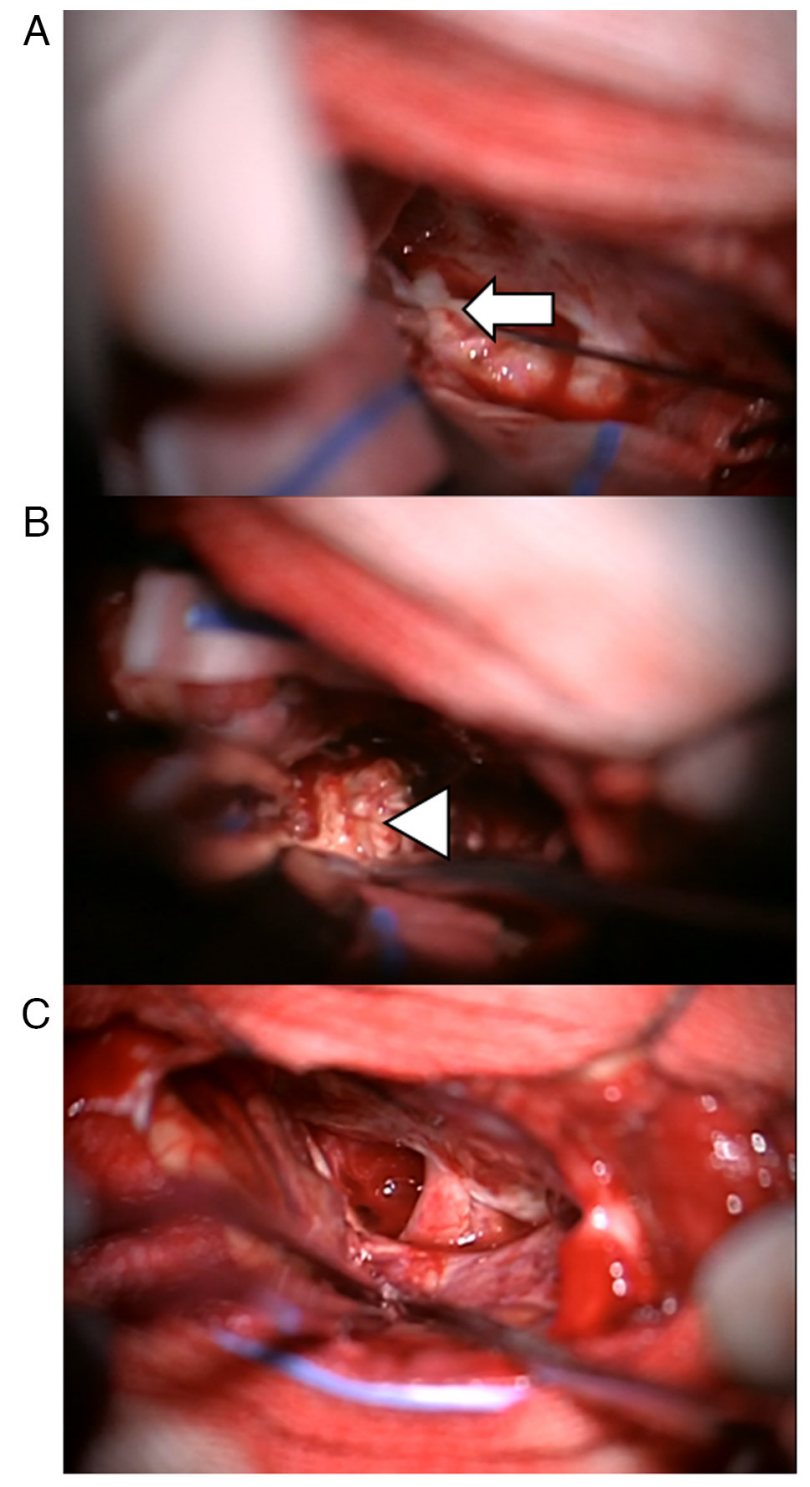
Intraoperative condition. (A) The wall of the capsule was opened during the operation to release the fluid, which was white and sticky (indicated by an arrow). (B) The lesion envelope (indicated by an arrowhead) was isolated along the periphery of the brain tissue. (C) After the lesion was removed, the pus was fully drained until the flushing solution was cool.

The surgery was performed via a right frontotemporal approach, revealing a grayish-white cystic-solid lesion extending from the right frontal base to the sellar region. Intraoperatively, thick, white, purulent fluid was aspirated from the cystic component, confirming an abscess (Fig. 4). The cystic cavity was irrigated with antibiotic solution and the surrounding solid lesion was meticulously excised. The excised specimen measured ~20x28x22 mm.

### Postoperative findings and follow-up

**Figure 5:**
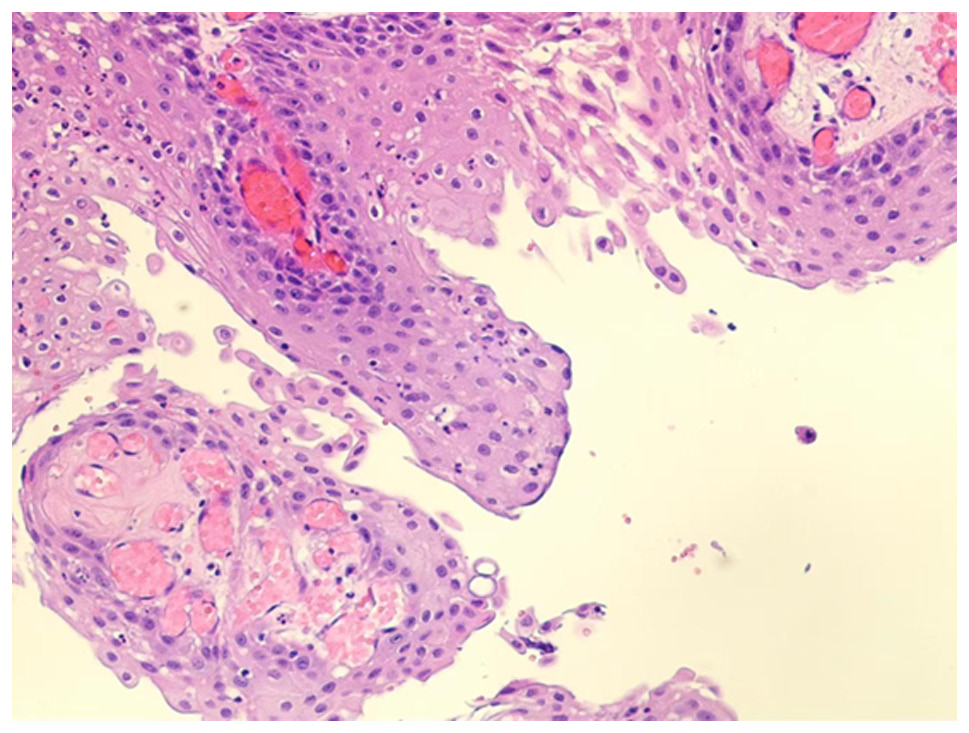
Histopathological examination of the resected tumor. Hematoxylin and eosin-stained section of the lesion wall reveals stratified squamous epithelium with papillary architecture, consistent with features of papillary craniopharyngioma. Marked inflammatory cell infiltration is also observed (magnification, x200).

**Figure 6:**
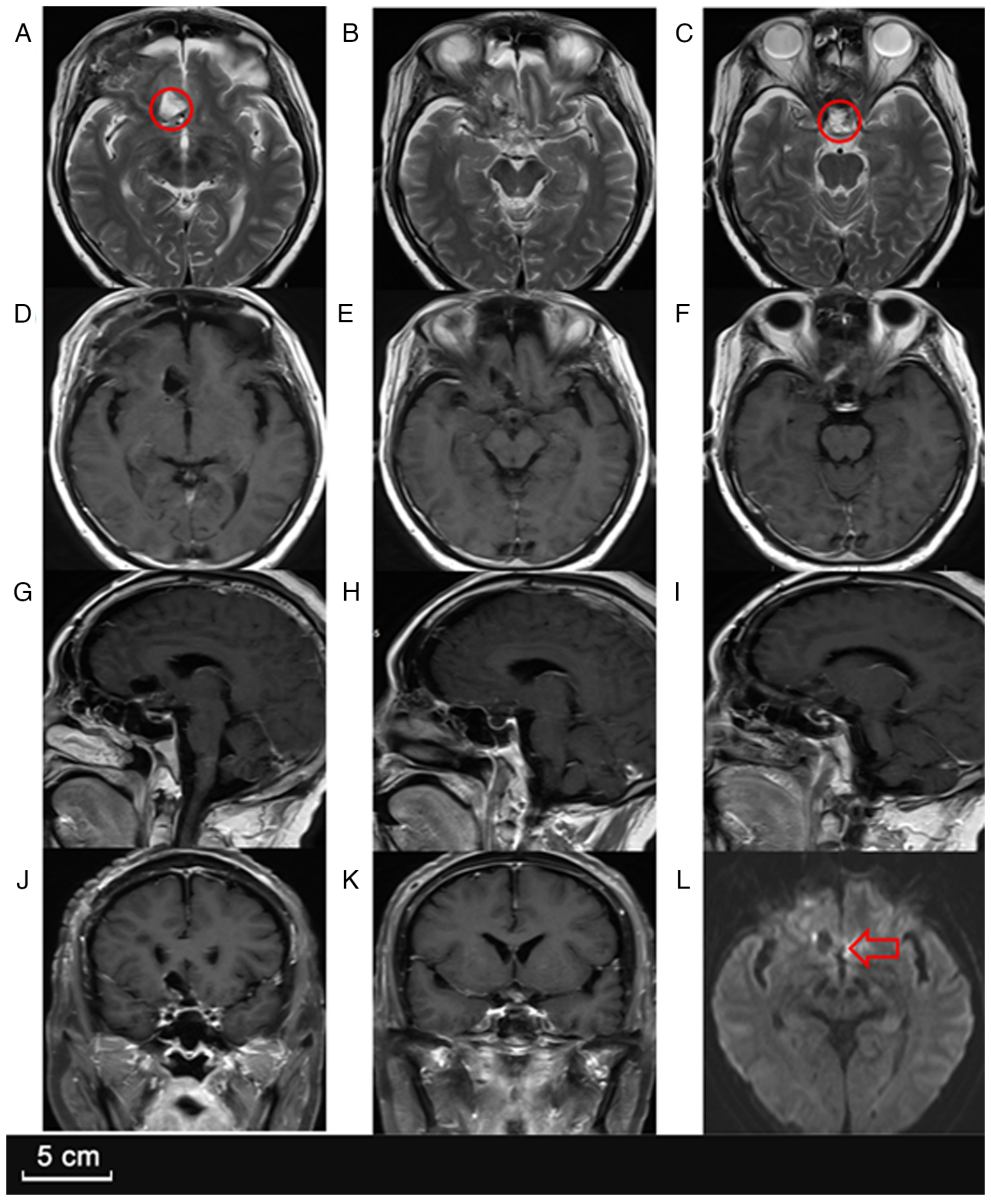
Postoperative MRI. (A-C) T2-weighted images showing high signal intensity in the (A) suprasellar (indicated by a circle) and (C) sellar (indicated by a circle) regions, consistent with cerebrospinal fluid. (D-K) Contrast-enhanced T1-weighted images showing no marked enhancement in the operative area. (L) Diffusion-weighted images indicating that the lesion had been completely removed and the pus had been drained (indicated by the arrow).

Tissue samples from the wall of the sellar abscess were fixed in 10% neutral-buffered formalin at room temperature (~22˚C) for 24 h. The fixed tissues were then processed and embedded in paraffin. Sections were cut at a thickness of 4 µm using a microtome. Routine hematoxylin and eosin staining was performed at room temperature, with hematoxylin staining for 5 min and eosin for 2 min. The stained slides were examined using a light microscope (Olympus BX53; Olympus Corporation). Images were captured at x200 magnification. A targeted next-generation sequencing approach (BGI Genomics Co., Ltd.) was used to detect a BRAFV600E mutation in the tumor sample. Genomic DNA was extracted from formalin-fixed, paraffin-embedded (FFPE) tumor tissue using the QIAamp DNA FFPE Tissue Kit (cat. no. 56404; Qiagen, Inc.) according to the manufacturer's protocol. DNA quantity and quality were assessed using a Qubit 4.0 Fluorometer (Thermo Fisher Scientific, Inc.) and an Agilent 2100 Bioanalyzer (Agilent Technologies, Inc.). Library preparation was conducted using the MGIEasy Universal DNA Library Prep Set (cat. no. 1000006985; BGI Genomics Co., Ltd.) following the manufacturer's instructions. Sequencing was performed on the MGISEQ-2000 platform with paired-end 150 base pair reads (PE150). The sequencing kit was the MGISEQ-2000RS High-throughput Sequencing Set (FCL PE150; cat. no. 1000005267; BGI Genomics Co., Ltd.). The final library was loaded at a concentration of 10 pM, quantified using a Qubit Fluorometer and calculated based on molar concentration. Raw sequencing data were processed with SOAPnuke (v1.5.6, https://github.com/BGI-flexlab/SOAPnuke) for quality control, and downstream analysis, including alignment, variant calling and annotation, was performed using Sentieon DNAseq (v202112.05; https://www.sentieon.com/), following the standard bioinformatics pipeline for BGI Genomics Co., Ltd. The histological and genetic analysis confirmed the presence of a papillary craniopharyngioma with a BRAFV600E mutation, accompanied by a marked inflammatory infiltrate (Fig. 5). Microbial cultures from intraoperative specimens were negative; however, next-generation sequencing (NGS) identified a Gram-positive *Staphylococcus* species, confirming a secondary pituitary abscess. Immediate postoperative MRI (Fig. 6) revealed complete resection of the lesion and a marked reduction in the mass effect. A follow-up hormone level assessment (Table I) was performed, confirming persistent hypopituitarism. The patient continued on antibiotic therapy (1,000 mg ceftriaxone, every day, intravenously for 3 weeks), levetiracetam (500 mg, twice per day, orally; continued postoperatively for prophylactic purposes) for seizure control, and hormone replacement therapy (50 µg levothyroxine, every day, orally; 10 mg prednisone, every day, orally; long-term postoperative replacement therapy was maintained until pituitary function recovered). The drainage tube was removed on the fifth postoperative day.

At the 3-month follow-up, the patient's vision had markedly improved, with better peripheral vision and reduced visual field defects. However, long-term hormone replacement therapy, including hydrocortisone, levothyroxine and desmopressin, was still required to manage the pituitary insufficiency and diabetes insipidus. The patient remains under regular follow-up (initially once a month, and then once every 3 months.) to monitor endocrine function and assess for potential recurrence.

## Discussion

Pituitary abscesses are rare in clinical practice and poorly understood. Pituitary abscesses are classified as either primary or secondary, depending on the presence or absence of underlying pituitary lesions. Secondary pituitary abscesses can occur in association with pre-existing conditions such as craniopharyngiomas, Rathke's cleft cysts and pituitary adenomas (8).

In a systematic review of 488 cases of pituitary abscesses, Stringer *et al* (6) found that 68% were primary and 32% were secondary. Secondary infections can be further divided into iatrogenic infections following surgery and spontaneous infections. Iatrogenic infections may be caused by a disruption of the normal blood supply to the pituitary, leading to decreased immune function in the sellar region (9), or by retrograde infection from sphenoid sinusitis (10,11). In addition to iatrogenic infections, there are a number of reports (2,12-14) of pituitary abscesses secondary to pre-existing sellar lesions, accounting for ~37% of secondary pituitary abscesses (15). This situation is the primary issue that the present study aims to discuss in relation to the present patient.

The English research literature was searched for isolated case reports of pituitary abscesses secondary to sellar lesions. A systematic literature search was performed using the PubMed database. The following search terms were used: ['pituitary abscess'(All Fields) OR 'sellar abscess'(All Fields)] AND ['case report'(Title/Abstract) OR 'case series'(Title/Abstract)] AND ['secondary'(Title/Abstract) OR 'sellar lesion'(Title/Abstract) OR 'sellar mass'(Title/Abstract) OR 'sellar tumor'(Title/Abstract)]. These scattered cases suggested that the pathophysiological mechanisms of such abscess formation are poorly understood (Table II) (2,9,13-47).

Pituitary abscesses are rare but serious complications, often arising as secondary infections in the context of underlying sellar lesions, including craniopharyngiomas. The pathophysiology behind secondary pituitary abscesses is multifactorial. Craniopharyngiomas, being locally invasive tumors, may cause significant inflammatory responses, leading to tumor necrosis and subsequent abscess formation. Additionally, the disruption of vascular supply to the pituitary gland due to the tumor's encroachment can compromise immune defenses, further predisposing the patient to infection (45,48). In the present case, the patient's craniopharyngioma exhibited extensive inflammatory infiltration, which likely contributed to the development of the abscess. A retrograde infection from adjacent structures, such as the sphenoid sinus or the nasopharynx, cannot be ruled out as a contributing factor. In the present case, the MRI findings suggested a mixed cystic-solid lesion in the suprasellar region, which was consistent with the craniopharyngioma (13). While the role of tumor-induced necrosis has been implicated in other reports, immune dysfunction plays a significant role in increasing the risk of secondary infections in these patients. In the present case, the inflammatory changes seen in the craniopharyngioma likely compromised local immune responses, predisposing the patient to the development of an abscess.

Clinically, secondary pituitary abscesses often present with symptoms related to the mass effect of the primary lesion and signs of infection. The most common non-specific symptoms include headache (91.7%) (8). Blurred vision and visual field defects (58%) (49) are the second most frequent symptoms, following headache. In addition to the mass effect, inflammation of the optic nerve and optic chiasm due to the abscess can also contribute to visual disturbances. Moreover, pituitary hormone secretion may be impaired due to the involvement of the pituitary gland, leading to hypopituitarism, with panhypopituitarism being the most common manifestation. The extent of pituitary dysfunction can reflect the severity of the abscess (50). This feature can make it difficult to differentiate from non-functioning pituitary adenomas; however, the latter typically do not present with signs of infection. If the lesion involves the pituitary stalk, polyuria (50%) may occur, with a higher incidence than in pituitary adenomas (51). The lack of typical infectious symptoms aligns with the literature, where secondary pituitary abscesses often lack clear signs of infection, complicating the diagnosis (13).

Diagnosing secondary pituitary abscesses is often challenging, particularly when they are associated with pre-existing lesions, as demonstrated in the present case. The initial clinical presentation of visual disturbances, headaches and nausea can easily be misattributed to the primary craniopharyngioma itself or to other more common causes of sellar mass effects. Therefore, early suspicion and a comprehensive diagnostic approach are crucial in identifying this rare complication. In the present case, advanced imaging modalities, including MRI with contrast and CT scans, were employed. Imaging by CT typically shows an enlarged sella turcica and a low-density sellar mass, which does not significantly aid in diagnosis. The typical MRI appearance is a cystic or cystic-solid sellar mass that is hypointense or isointense on T1-weighted images and hyperintense or isointense on T2-weighted images. A characteristic finding is the rim enhancement of the cystic mass after contrast administration (52,53). Due to the restricted diffusion within the abscess cavity, it often appears hyperintense on diffusion-weighted imaging, which can help differentiate a pituitary abscess from other sellar lesions (54). However, there are reported cases that lack this feature (55,56).

According to reports, the most common causative microorganisms in secondary pituitary abscesses are gram-positive bacteria, including *Staphylococcus* and *Streptococcus* species. However, as in the present case, numerous patients have negative culture results whereby no pathogenic organisms are identified from secretions. In >50% of cases, pathogens fail to be isolated (57). This absence of bacteria may be related to the use of antibiotics before surgery or limitations of culture conditions (54-56). Thus, metagenomic NGS (mNGS) is recommended for analyzing abscesses and tissues obtained during surgery, as mNGS provides more accurate information on the presence of pathogens. The mNGS platform can perform high-throughput nucleic acid analysis of samples containing microorganisms and match them to reference genomes to identify the microbial species present and their relative abundance (57,58).

For the treatment of secondary pituitary abscesses, surgical intervention is considered the preferred approach (59). Surgery serves two primary purposes: It alleviates the mass effect of the primary lesion and promptly drains the infection, thereby preventing further progression of the infection and minimizing the risk of permanent neurological and pituitary dysfunction. Upon the identification of symptoms suggestive of a pituitary abscess, empirical antibiotic therapy should be initiated immediately to establish an optimal window for surgical intervention. This early antibiotic treatment is crucial in controlling infection and preparing the patient for subsequent surgery.

Regarding surgical options, the endoscopic transsphenoidal approach is generally the first choice due to its ability to reduce the risk of infection spread and minimize damage to critical structures such as the optic nerves. This minimally invasive technique offers a relatively quick recovery time and avoids the need for more invasive procedures. However, if the pituitary abscess extends into the suprasellar region or if the patient has contraindications for transnasal surgery (such as anatomical anomalies or prior nasal surgery), a craniotomy may be necessary for abscess drainage (60). Craniotomy, while effective, is associated with a higher risk of abscess recurrence (7), and requires more extensive postoperative care.

For the patient in the present case, the abscess had significantly extended into the right frontal lobe, prompting the decision to opt for craniotomy to ensure thorough and effective drainage. During surgery, the abscess wall was excised completely to minimize the risk of recurrence and repeated irrigation of the surgical site was performed with saline and antibiotics to further reduce the infection risk.

The timing of surgery is also critical. Procedures should be avoided during the acute phase of infection or when the patient presents with a high fever, as this can increase the risk of bacteremia and sepsis. During this period, the patient should receive appropriate antibiotic therapy and fluid support to manage symptoms. The selection of antibiotics should be tailored to the identified or suspected pathogen, with consideration for local antibiotic resistance patterns (61). Emerging clinical guidelines emphasize the importance of initiating broad-spectrum antibiotics until culture or sequencing results are available, followed by a more targeted regimen based on these findings (62).

Postoperatively, antibiotic therapy should continue for 3-4 weeks, adjusted according to the results of culture or sequencing. Although some patients may experience partial recovery of pituitary function following surgery (32.3%) (7), hormone replacement therapy remains essential. Long-term management is particularly important for patients who had significant preoperative pituitary dysfunction, as these individuals are at a high risk of developing permanent hypopituitarism after surgery (59).

Usually, it is difficult to identify the primary lesion in secondary pituitary abscesses based solely on the patient's history and imaging before surgery. Only after obtaining histopathological evidence during surgery can clinicians determine the primary sellar lesion, although histopathology often does not fully explain the source of the sellar infection. An early and accurate diagnosis of a pituitary abscess, followed by timely intervention, can lead to a better prognosis (63).

A comprehensive review of the literature highlights that secondary pituitary abscesses are more commonly associated with pituitary adenomas (64%) and Rathke's cleft cysts (24%) than with craniopharyngiomas (12%). Despite the relatively low frequency of craniopharyngioma-associated abscesses, this case illustrates the significant differential diagnostic challenges that clinicians face. Differential diagnoses for secondary pituitary abscesses include other sellar lesions such as pituitary adenomas, Rathke's cleft cysts and sellar metastases. Inflammatory conditions, such as sarcoidosis or tuberculosis, should also be considered, as these can mimic the clinical and radiological features of a pituitary abscess (64). A detailed clinical history, coupled with advanced imaging and microbiological workup, is essential for distinguishing these entities.

The present case report had several limitations. The lack of long-term follow-up is a significant limitation, as the resolution of abscesses and recovery of pituitary function can evolve over time. Additionally, the rarity of secondary pituitary abscesses means that our understanding of the optimal treatment regimen is still evolving. Further investigation, ideally through multi-center studies, is necessary to establish consensus guidelines for the management of this rare and challenging condition.

In conclusion, the present case highlighted the rare occurrence of a secondary pituitary abscess arising from a craniopharyngioma, emphasizing the diagnostic and therapeutic challenges associated with such cases. Given the overlapping clinical and radiological features with other sellar lesions, maintaining a high index of suspicion is crucial for early identification. A multidisciplinary approach involving neurosurgeons, endocrinologists and infectious disease specialists is essential to ensure prompt diagnosis and tailored management. The present literature review provided a more comprehensive discussion of the pathophysiology, clinical implications and management strategies for this rare entity. The importance of early surgical intervention combined with appropriate antibiotic therapy to optimize patient outcomes was underscored. Furthermore, long-term follow-up is necessary to monitor endocrine function and prevent recurrence. By detailing this case and analyzing the existing literature, the present study aimed to enhance awareness among clinicians regarding the potential for secondary pituitary abscesses in patients with pre-existing sellar lesions, ultimately contributing to improved diagnostic accuracy and patient care.

## Figures and Tables

**Table I:** Patient's laboratory results and normal ranges.

Test	Pre-surgery result	Post-surgery result (1 week after surgery)	Normal range
ACTH, pg/ml	5.34	5.07	0-46
IGF-1, ng/ml	33.7	72.4	45-210
Free thyroxine, pmol/l	7	12.1	7.64-16.03
TSH, µIU/ml	1.62	<0.005	0.49-4.91
FSH, mIU/ml	1.89	<0.1	0.7-11.1
LH, mIU/ml	<0.1	<0.1	0.8-7.6
Progesterone, ng/ml	<0.2	<0.2	0.27-0.90
Testosterone, ng/ml	<0.2	<0.2	2.1-7.5 (male >50 years)
Estradiol, pg/ml	<11.8	<11.8	0-39.8 (male)
Prolactin, ng/ml	54.4	<0.5	2.5-17
Urine specific gravity	1.002	1.002	1.003-1.030
24-h urine sodium content, mmol/24 h	609.96	458.65	40-220

ACTH, adrenocorticotropic hormone; IGF-1, insulin-like growth factor 1; TSH, thyroid-stimulating hormone; FSH, follicle-stimulating hormone; LH, luteinising hormone.

**Table II:** Reported cases of secondary sellar infections following sellar lesions (1952-2024).

First author, year	Cases, n	Pathological diagnosis	Culture	Treatment	Outcome	(Refs.)
Whalley, 1952	1	Pituitary adenoma	*Escherichia coli*	None	Died before treatment	(16)
De Villiers Hammann, 1956	1	Pituitary adenoma	*Staphylococcus aureus*	Transcranial surgery	Recovered	(17)
Obenchain and Becker, 1972	1	Rathke's cleft cyst	*Staphylococcus aureus*	Transcranial surgery	Recovered	(18)
Obrador and Blazquez, 1972	1	Craniopharyngioma	Negative	Transcranial surgery	Recovered	(19)
Domingue and Wilson, 1977	2	Pituitary adenoma	1 negative; 1 *Diplococcus pneumoniae*	1 Transcranial surgery; 1 transnasal surgery	1 died after recurrence; 1 died after sepsis	(20)
Zorub *et al*, 1979	1	Pituitary adenoma	*Streptococcus pneumoniae*	Transnasal surgery	Died after acute hydrocephalus	(21)
Gomez Perun *et al*, 1981	1	Rathke's cleft cyst	NS	Transcranial surgery	NS	(22)
Holck and Laursen, 1983	1	Pituitary adenoma	Negative	Transnasal surgery	NS	(23)
Nelson *et al*, 1983	3	Pituitary adenoma	*Bacteroides*	1 transcranial surgery; 2 transnasal surgery	3 recovered	(24)
Bossard *et al*, 1992	1	Pituitary adenoma	NS	Transnasal surgery	NS	(25)
Bognàr *et al*, 1992	2	Rathke's cleft cyst	*Staphylococcus aureus* and *Streptococcus pyogenes*	1 transcranial and transnasal surgery; 1 transnasal surgery	1 died after secondary surgery; 1 recovered	(26)
Shanley and Holmes, 1994	1	Craniopharyngioma	*Salmonella typhi*	Transnasal surgery	Recovered	(27)
Jain *et al*, 1997	2	1 Rathke's cleft cyst, 1 pituitary adenoma	1 *negative; 1 Aspergillus*	1 transcranial surgery, 1 transnasal surgery	2 recovered	(28)
Thomas *et al*, 1998	1	Rathke's cleft cyst	Anaerobic streptococci	Transnasal surgery	NS	(29)
Jadhav *et al*, 1998	1	Pituitary adenoma	*Staphylococcus epidermidis*	Transcranial surgery	Recovered	(30)
Sharma *et al*, 2000	1	Pituitary adenoma	Tuberculosis	NS	Recovered	(31)
Kroppenstedt *et al*, 2001	1	Pituitary adenoma	Negative	Transnasal surgery	Recovered	(32)
Vates *et al*, 2001	5	3 pituitary adenoma, 1 craniopharyngioma; 1 rathke's cleft cyst	2 negative; 1 *Streptococcus pneumoniae*; 1 *Staphylococcus aureus*; 1 alpha *Streptococcus*	NS	NS	(9)
Zhang *et al*, 2002	1	Pituitary adenoma	*Toxoplasma gondii*	NS	NS	(33)
Jaiswal *et al*, 2004	1	Pituitary adenoma	*Escherichia coli*	Transnasal surgery	Recovered	(34)
Hatiboglu *et al*, 2006	1	Pituitary adenoma	Gram-positive cocci	Transnasal surgery	Recovered	(35)
Dutta *et al*, 2006	1	Pituitary adenoma	MRSA	Transnasal surgery	Recovered	(36)
Celikoglu *et al*, 2006	2	Rathke's cleft cyst	NS	Transnasal surgery	2 recovered	(37)
Takayasu *et al*, 2006	1	Rathke's cleft cyst	*Pseudomonas aeruginosa*	Transnasal surgery	Recovered	(38)
Salinas-Lara *et al*, 2008	1	Pituitary adenoma	Mucormycosis	Transnasal surgery	Died during surgery	(39)
Ciappetta *et al*, 2008	1	Pituitary adenoma	Negative	Transnasal surgery	Recovered	(15)
Qi *et al*, 2009	1	Craniopharyngioma	*Staphylococcus aureus*	Transcranial surgery	Recovered	(40)
Bakker and Hoving, 2010	1	Pituitary adenoma	MRSA	Transcranial surgery	Recovered	(41)
Kuge *et al*, 2011	1	Pituitary adenoma	Gram-positive cocci	Transnasal surgery	Recovered	(42)
Kotani *et al*, 2012	1	Pituitary adenoma	Gram-negative cocci	None	Died	(43)
Awad *et al*, 2014	4	Pituitary adenoma	1 group A *Streptococcus*; 1 *staphylococcus epidermidis*; 1 MRSA; 1 *Streptococcus pneumoniae*	Transnasal surgery	3 recovered; 1 died	(14)
Safaee *et al*, 2016	1	Pituitary adenoma	*Staphylococcus aureus*	Transnasal surgery	Recovered	(2)
Muscas *et al*, 2017	1	Pituitary adenoma	Negative	Transcranial surgery	Died after surgery	(44)
Bhaisora *et al*, 2018	1	Craniopharyngioma	*Staphylococcus aureus*	Transnasal surgery	Recovered	(45)
Coulden *et al*, 2022	1	Rathke's cleft cyst	*Staphylococcus aureus*	Transnasal surgery	Recovered	(46)
López Goméz *et al*, 2022	1	Caniopharyngioma	Negative	Transnasal surgery	Recovered	(13)
Inoue *et al*, 2024	1	Rathke's cleft cyst	Negative	Transnasal surgery	Recovered	(47)

NS, items not explicitly stated in the original literature; MRSA, methicillin-resistant *Staphylococcus aureus*.

## Data Availability

The data generated in the present study may be requested from the corresponding author.
